# Barriers to using postpartum family planning among women in Zanzibar, Tanzania

**DOI:** 10.1186/s12905-023-02330-2

**Published:** 2023-04-17

**Authors:** Kristina Söderbäck, Herborg Holter, Sanura Abdulla Salim, Helen Elden, Malin Bogren

**Affiliations:** 1 Sahlgrenska Academy, University of Gothenburg, Gothenburg, Sweden; 2 Institute of Health and Care Sciences, University of Gothenburg, Gothenburg, Sweden; 3 Mnazi Mmoja Hospital, Zanzibar, Tanzania

**Keywords:** Contraceptive methods, Low-income country, Maternal health, Postpartum family planning, Sexual reproductive rights, Sub-Saharan Africa

## Abstract

**Background:**

Effective family planning is associated with substantial benefits, including reductions in maternal and neonatal mortality due to the avoidance of unintended pregnancies, and contributions to spacing, timing, and limiting births. However, in Zanzibar, Tanzania, the utilization of modern contraceptive methods is low. This study therefore aimed to identify barriers to using postpartum family planning among women in Zanzibar.

**Methods:**

Five focus group discussions were conducted with 24 women who gave birth in the maternity unit at a reference hospital in Zanzibar during the first quarter of 2022. The discussions took place in Swahili, were performed with the assistance of an interview guide, and were audio recorded, transcribed in Swahili, and translated to English. Data were analysed with qualitative content analysis using an inductive approach.

**Results:**

Barriers to using postpartum family planning in Zanzibar could be summarized in three generic categories. *Inadequate knowledge about postpartum family planning* is expressed in the subcategories: inadequate knowledge about contraceptive methods and their mode of action, insufficient quality of family planning services, and belief in traditional and natural medicine for family planning. *Perceived risks of modern contraceptive methods* are described in the subcategories: fear of being harmed, and fear of irregular bleeding. *Limited power in one’s own decision about contraceptive use* consist of the subcategories: the need to involve the husband, and opposition and lack of interest from the husband.

**Conclusions:**

The participants’ current knowledge of postpartum family planning was insufficient to either overcome the fear of side-effects or to understand which side-effects were real and likely to happen. The woman’s power in her own decision-making around her sexual reproductive rights is of critical importance. Given the barriers identified in this study, the findings call for increased knowledge about family planning methods and their mode of action, and involvement of the husband throughout pregnancy, childbirth, and the postpartum period in postpartum family planning education and counselling, in Zanzibar and in similar settings.

**Supplementary Information:**

The online version contains supplementary material available at 10.1186/s12905-023-02330-2.

## Background

Increased access to family planning, including postpartum family planning, is recognized as a critical need if universal health coverage is to be achieved; that is, having the full spectrum of quality health services and access to sexual and reproductive healthcare [1]. The significant benefits associated with effective family planning are clear, with reductions in.

maternal and neonatal mortality due to the avoidance of unintended pregnancies, and contributions to spacing, timing, and limiting births [2, 3]. When couples are enabled to plan their future childbirths, girls have a better chance of completing their education, which in turn strengthens the economic security and well-being of their families [4]. Effective family planning thus helps countries work toward three of the UN’s Sustainable Development Goals (SDG): good health and well-being (SDG 3), quality education for all (SDG 4), and gender equality (SDG 5) [4, 5].

The annual figure of 111 million unintended pregnancies in low- and middle-income countries reflects an unmet need for family planning including postpartum family planning among women. It is a global challenge to address this need among women who want to avoid pregnancy but who are not using any contraceptive method or who use ineffective traditional contraceptive methods [6–8]. This also a challenge in sub-Sahara Africa, were only 55% of the need for family planning is being met with modern methods [9]. In Tanzania, only 33% of all married women between 15 and 49 use a modern contraceptive method, and the unmet need for contraception has stagnated at a level of 22–24% over the past two decades [10]. A recent study from Tanzania reports that the median time for resuming sexual activity after childbirth was 2 months, while the median time for starting contraceptive use was 7 months. The five months gap means that the women postpartum are at high risk of getting unintended pregnancies [11]. According to the latest statistics from 2015 to 2016, Zanzibar, a semi-autonomous part of Tanzania, has a total fertility rate of 5.1 children per woman and 25.4% of all children were born within 23 months of the previous childbirth [10]. Maternal mortality is 267 out of 100 000 live childbirths, and under-5-years mortality is 56 deaths out of 1 000 live childbirths; newborn death accounts for 50% of the latter figure. [12]. Currently married women in Tanzania are more likely to use modern contraceptive methods (33%) than women in Zanzibar (14%). However, there are large variations across regions within Zanzibar ranging from 7 to 29% [10]. Although postpartum family planning are provided by the government’s health facilities for free [12], the total demand for general family planning among married women aged 15–49 in Zanzibar was 51% in 2016 and the unmet need was 28% [10]. The number of postpartum family planning cases seem to be low (12) and there are to our knowledge no scientific studies conducted to understand the situation and what factors contributing to this trend.

Previous studies conducted in Zanzibar show that there is a low utilization of modern contraceptive methods [10, 13, 14]. This study was therefore intended to fill a knowledge gap regarding specific factors which create barriers to using postpartum family planning in Zanzibar. The aim of the study was to explore barriers contributing to the low utilization of postpartum family planning by women at a reference hospital in Zanzibar. The results from this study are presumed to be useful also in similar settings intending to improve uptake of postpartum family planning.

## Methods

### Study design

A qualitative research design was used, with data collected in focus group discussions (FGDs) [15] among women who had given birth within 1–3 days previously. The research questions were: What are the most prominent barriers for use of postpartum family planning in Zanzibar? What do women in Zanzibar need from family planning services, and how can those needs be met? The study was approval from the Zanzibar Health Research Ethical Committee, Zanzibar Health Research Institute (ref: ZAHREC/04/PR/FEB/2022/03).

#### Study setting

The study took place at the maternity clinic at a reference hospital in Zanzibar. In 2020, 13 584 childbirths were recorded but only 1.19% of the mothers utilized the family planning services at the hospital. Counselling about postpartum family planning starts during the antenatal visits and continues during the postnatal care. Attendance at antenatal visits is low, and only 43% of all mothers attend the recommended postnatal visits within 42 days after childbirth in Zanzibar [12].

### Participants and recruitment

Women who had recently given birth and were inpatients at the maternity ward during the time of data collection were eligible for this study. The inclusion criteria were a pregnancy and childbirth without complications, age above 18 years, and a woman in overall good health. The recruitment of eligible women was based on a convenience sampling, took place face-to-face in the maternity ward by a native-speaking midwife. A total of 24 women participated in the study, including both those who had given birth vaginally and those who had undergone caesarean section. Details of their demographic characteristics are given in Table 1. Some women declined participation because they would have to move from their bed, which they thought might cause discomfort; others declined due to the need to look after their newborn; and some declined simply because they were tired after the childbirth. The number of women who declined to participate was not calculated.

**Table 1:** Demographics of the participants (n = 24)

	n	%
**Age (years)**		
20–24	3	12.5
25–29	11	45.8
30–34	4	16.7
35–44	5	20.8
Missing data	1	4.2
**Number of children**		
1–2	7	29.2
3–4	13	54.1
5–6	4	16.7
**Relationship status**		
In a relationship but not married	1	4.2
Married	22	91.6
Widow	1	4.2
**Completed education level**		
Primary school (7 years)	4	16.7
Secondary school	16	66.6
University	3	12.5
Missing data	1	4.2
**Religion**		
Islam	22	91.6
Christianity	1	4.2
Other	1	4.2
**Ever tried a modern contraceptive method**		
Yes	14	58.3
No	9	37.5
Missing data	1	4.2

### Data collection methods

Data were collected through five audio recorded FGDs during the first quarter of 2022. All participants gave their written consent to participate after having received verbal and written information about the study, including the fact that participation was voluntary and that they had the right to withdraw at any time without explanation. The FGDs were conducted in a separated room near the maternity clinic. Age variation and parity were not considered when constituting the groups. All FGDs were conducted in Swahili by one of the authors (SS) and followed an interview guide (Appendix 1). The interview guide contained an overall question: *Given that you just gave birth, have you considered using any family planning to delay your next pregnancy?* Probing questions such as *Can you explain why?* were asked. The interview guide was piloted with one FGD, which provided rich content and thus was included in the analysis. Each FGD contained 3–6 women and lasted for 28–52 min (mean: 40 min).

### Data analysis processes

The interviews were translated verbatim into English. The transcripts were then analysed using the principles of inductive content analysis as described by Elo and Kyngäs [16]. First, the text was read several times to make sure no information was missing and to make sense of the information as a whole. Next, meaning units were identified which answered the research questions (‘What are the most prominent barriers for use of postpartum family planning?’ and ‘What do the women need from family planning services, and how can those needs be met?’). The meaning units were then compared and sorted into codes based on similar content, which were thereafter compared and clustered into generic categories. This analysis was conducted by KS, in close collaboration with MB and SS, until full agreement was reached. The final phase of the analysis involved all authors. Examples of the analytical process are given in Table 2.

**Table 2:** Examples of the analytical process

Meaning unit	Code	Subcategory	Generic category
*No, I can’t put an intrauterine contraceptive device or implant in before I consult my spouse. He’ll tell me I despise him. You don’t know men these days, suddenly he tells you to take your divorce. (FGD3)*	The woman needs to involve her husband	The need to involve the husband	Limited power in one’s own decision about contraceptive use
*Yeah! It’s really, it’s cancer, because you get an implant and it disappears before you plan to remove it. Nobody knows where it’s going, so it’s the source of cancer.* (FGD1)	The woman believes that the implant causes cancer	Fear of being harmed	Perceived risks of modern contraception methods

## Results

Barriers to using postpartum family planning are presented in three generic categories, each with two or three subcategories; see Table 3 for an overview. Quotations from the five FGDs are labelled 1–5.

**Table 3:** Generic categories and subcategories illustrating barriers to using postpartum family planning

Generic category	Subcategory
Inadequate knowledge about postpartum family planning	Inadequate knowledge about contraceptive methods and their mode of action
	Insufficient quality of family planning services
	Belief in traditional and natural medicine for family planning
Perceived risks of modern contraceptive methods	Fear of being harmed
	Fear of irregular bleeding
Limited power in one’s own decision about contraceptive use	The need to involve the husband
	Opposition and lack of interest from the husband

### Inadequate knowledge about postpartum family planning

Inadequate knowledge about postpartum family planning methods included three subcategories: inadequate general knowledge about contraceptive methods and their mode of action, insufficient quality family planning services, and belief in traditional and natural medicine for family planning.

#### Inadequate knowledge about contraceptive methods and their mode of action

A prominent barrier to using postpartum family planning was women’s inadequate knowledge about the different contraceptive methods and their mode of action. This was reflected by the women’s questions about where in the body the intrauterine contraceptive device was placed, how and when it could be removed, whether a woman could get pregnant with it in place, and so on. The women did know about the benefits of postpartum family planning, such as it giving time for the mother to recover and the baby to grow up healthy, but none of them could explain the contraceptive methods’ mode of action in the body. Moreover, limited knowledge about female reproductive health organs and the body was revealed by the women’s explanations and worries about the implant or intrauterine contraceptive device getting lost and circulating in the body.

*As far as I know, when you use contraception, you restore your health as well as that of your baby, but I don’t understand the contraceptive methods. (FGD2)*

#### Insufficient quality of family planning services

Insufficient quality of family planning services was another barrier to using postpartum family planning. Low healthcare quality seemed to have contributed to poor, incomplete, and sometimes incorrect knowledge about contraception. For example, one explanation from healthcare professionals for why an implant in the arm could not be found by palpation was that it had melted or vanished. The women had experiences with doctors and other healthcare professionals who did not give them the clear information they needed to reach understanding. The participants emphasised the need for extended information from the healthcare facilities about the contraceptives’ mode of action and side-effects.

*(…) the doctors in the health facilities don’t give you satisfactory answers. You need more explanation to be satisfied with their advice. But their answers are very brief. (FGD5)*

Because of the insufficient quality of family planning services, there was doubt among the women regarding whether the contraceptive methods would be effective. This doubt also came from stories from others, and from the women’s own experiences, of becoming pregnant despite using contraceptives. The reasons for this were either that the woman was already pregnant when she began using the contraceptive method, or that the intrauterine device was not inserted correctly; both of these were caused by insufficient healthcare quality.

*I was thinking about joining the family planning and I used the loop method, but I was disappointed. After four months I found out I was pregnant while using the intrauterine device. The next day I went back to the hospital, actually, I didn’t like his answer. He said that it was improperly inserted. So disappointed!* (FDG1)

The insufficiency of quality family planning services was also reflected by the fact that few of the women knew about postpartum family planning services, and that they could receive these before discharge from the maternity ward. This resulted in women feeling unprepared and unable to decide about contraceptive use when it was offered in the maternity ward.

#### Belief in traditional and natural medicine for family planning

There was widespread belief in traditional postpartum family planning methods such as breastfeeding, as well as traditional medicine. As breastfeeding delayed the return of menstruation, the women said that they planned to breastfeed for two years and that they felt that this was a safe contraceptive method. Breastfeeding was also described as an accepted method in the Quran.

*I will only use contraception when I have completed two years of breastfeeding, to keep my baby healthy.* (FGD2)

The women expressed strong belief in traditional and natural medicine and its methods as effective and safe. They said that they commonly used traditional medicine such as swallowing black seeds and wearing leaves around the hips for preventing pregnancy, as some believed these methods to be helpful in avoiding a new pregnancy.

*I use natural remedies, like swallowing black seeds, that are very helpful.* (FGD2)

However, other women who had used these traditional methods said that they later found themselves pregnant. Other examples of traditional contraceptive methods included the calendar method and putting trust in God. Overall, these contraceptive methods were barriers to seeing the usefulness of modern contraceptive methods.

### Perceived risks of modern contraceptive methods

Perceived risks from using contraceptives included two subcategories: fear of being harmed (in general, or even the risk of becoming infertile) and fear of irregular bleeding.

#### Fear of being harmed

The fear of being harmed using contraception was related to worries about increased weight, increased heart rate, high blood pressure, genital infections, genital cancer, a swollen abdomen, and increased vaginal discharge. These symptoms and illnesses were not always seen as being clearly the side-effects of contraceptive methods, as the women were not always sure that their contraceptive method was causing their symptoms; but the symptoms created a fear of using the contraceptives. Other women stated that the risk of getting these symptoms and illnesses depended on each woman’s unique body.

*Local discussions are an obstacle that keeps us from taking contraception. Some people talk about this, and some people talk about that, from the people who used the family planning method. So you’re afraid; you feel that you’ll end up being harmed like them.* (FGD2)

Another barrier to using postpartum family planning was the fear of damage to the uterine tissues due to the influence of mechanical factors, causing difficulty in becoming pregnant or even infertility. The risk of delay in conceiving the first pregnancy contributed to this fear, but so did stories about other women’s experiences of having trouble conceiving after using contraceptive methods.

*…but most importantly, people who complain a lot say that using contraception causes the uterus to become soft and unstable. That’s why many people who use contraception, after they stop using it and try to conceive, they often miscarry.* (FGD4)

#### Fear of irregular bleeding

The fear of irregular or heavy bleeding was another barrier to using postpartum family planning. Some women had stopped using contraception because of irregular bleeding patterns or absence of menstruation, not knowing that some contraceptive methods had that effect. They said that a regularity in their monthly menstruation assured them that their body was healthy, while the absence of it made them worry that they might be pregnant.

*I’m afraid of using contraception, and because I have no bleeding I won’t know if it will affect me or not.* (FGD4)

### Limited power in one’s own decision about contraceptive use

The women’s limited power in making their own decisions about contraceptive use included two subcategories: the need to involve the husband, and opposition and lack of interest from the husband.

#### The need to involve the husband

Using postpartum family planning meant that the woman needed to involve her husband and get his permission to use contraceptives. If she decided on her own to use contraceptives, this often led to misunderstandings. In some cases, the husband could even interpret it as her despising him because she wanted to avoid a new pregnancy, or as her being unfaithful to him.

*No, I can’t put [an intrauterine contraceptive device or implant] in before I consult my spouse. He’ll tell me I despise him. You don’t know men these days, suddenly he tells you to take your divorce.* (FGD3)

One woman described a complicated situation where she wanted to take the decision by herself to protect her and the children’s health, but because of her dependence on her husband, she needed to consult him to maintain peace in the marriage.

*For my part, I want to get my tubes tied and never give birth again. He told me that the decisions you’ve made, you haven’t made with me. I told him you see my condition is not good when I am pregnant, I’m suffering, he said we’ll talk. You really need to involve him, but on the other hand you must stand up as a woman just to defend yourself, because you’re the only one who’s hurting. Because he’ll go for another woman and have another child [with her].* (FGD3)

Most women planned to live in their parents’ house after childbirth, and to move back to their husband after at least 40 days; but in some cases, it could take up to six months. The reasons they gave for staying with their parents were to get some rest, and to avoid getting pregnant again. Thus, postpartum family planning was not considered an urgent matter after childbirth, but was desired later, when moving back to the husband. One woman said:

*The concern comes when your husband may need you for sex, so 40 days after giving birth, then you go back to your husband with your protection.* (FGD5)

It was the husband who decided when the woman moved back to him, as well as whether contraception should be used.

The women suggested that their husbands should be invited to the antenatal clinic together with them and receive education and information about postpartum family planning. Another suggestion was that the husband should visit his wife at the maternity ward and jointly discuss postpartum family planning. The women thought this discussion would be easier if their husbands had obtained trustworthy information from the healthcare professionals, leading to a better knowledge of the topic. In addition, an increased presence of the husband during and after labour and birth was thought to ease the couple’s decision-making about postpartum family planning.

*Don’t leave out the father. He should be involved because he’s the one who stops us from using family planning. He should be involved in the education about the benefits of family planning. And then we’ll sit together, husband and wife, to discuss it. (FGD2)*

#### Opposition and lack of interest from the husband

Opposition from the husband included threats of divorce, which made the women yield to their husbands’ opposition.

*When you want to use family planning, your husband threatens you, so you’re afraid, and you don’t make use of family planning, you give in to your husband’s threats.* (FGD1)

Because some men lacked interest in learning about and participating in family planning issues in general, it was difficult for the women to discuss the topic with their husbands, and to argue if the men were against using contraception. This situation caused anxiety among the women about getting pregnant unintentionally. According to the participants, one explanation for men’s opposition to family planning was that men took pride in having many children.

*Also, these men sitting on the porch, talking to each other as we women talk to each other. So, someone talking about how his wife is using family planning and starting a rumour, or sometimes they feel proud if their wife gives birth every year. Taking it very easy, and he feels like he’s better than other men.* (FGD4)

Some participants stressed the need to be independent in their own decision making to avoid short interval between pregnancies, and for the sake of the baby’s health. To not involve the husband and instead using contraceptive methods behind his back, was a way around a possible opposition, even though this could lead to negative consequences within the marriage.

## Discussion

This study from Zanzibar revealed several barriers which kept women from using postpartum family planning. Inadequate knowledge about postpartum contraceptive methods along with insufficient quality of family planning services created barriers, and belief in traditional and natural medicine was a barrier to seeing the usefulness of modern family planning. Perceived risks, and hearing about risks from other women, created a fear that contraceptive use would cause harm and irregular bleeding. Another barrier was the woman’s lack of power in her own decision-making about contraceptive use. These results mirror the most frequently given reasons for not using contraception among married women with unmet need in Tanzania [17], and are also in line with findings from other studies performed in low- and middle-income countries [18–20]. Hindrances to achieving sexual health and rights, including use of postpartum family planning, arise from several issues such as gaps in healthcare quality, as well as from social, cultural, and gender issues [21].

Among the women in the present study, a lack of knowledge about contraceptive methods and reproductive health was a barrier to making informed decisions about what method to use and to understanding the usefulness of modern contraceptive methods. Provision of quality family planning services require skilled healthcare professionals in combination with appropriate contraceptive methods [22]. It can be argued that Zanzibar, like any other developing country face shortage of essential qualified staff for family planning services. This can explain that women in this study did not receive necessary information about the contraceptives’ mode of action and side-effects. Unless interventions targeting healthcare professionals to improve quality and services by expanding contraceptive choices, counselling, and competency, family planning services will fail to reach out with accurate and understandable information.

That the women lacked knowledge about contraceptive methods and reproductive health can

also be a consequence of limited sexuality education in some cultures that makes it more difficult for women and men to understand reproductive health and contraceptive methods [7]. Studies have found that when sexuality education in reproductive health and rights is accessible in schools, in out-of-school programs, and in health facilities, there is a decrease in risky sexual behaviour and unintended pregnancies [7]. Countries investing in comprehensive family planning programs, including sexuality education with adequate information, education, and communication about family planning, have proven in the past to be successful, with increased demand and use of modern contraceptives and decreasing unmet need [23].

A critical barrier in this study was the fear of being harmed by side-effects. It was clear that the women did not always understand if a side-effect they had heard about was due to contraceptive methods and likely to happen, or a misconception. It is important to differentiate between real side-effects caused by the contraceptive methods and diseases or health states such as cancer or infertility, which are misunderstood as side-effects [19]. The fear of having an ineffective method could be a sign of mistrust in the healthcare services and in the contraceptive methods, which might be a consequence of insufficient healthcare quality. This fear can be overcome by introducing improved routines that aim to assure good quality, and better education among healthcare professionals working in family planning. It is therefore of high importance that these healthcare professionals give women and couples comprehensive information about different contraceptive methods, their modes of action, and their potential side-effects [17, 21].

This study showed that women in Zanzibar experienced a lack of power in their own decision-making about family planning. The understanding that the decision should be taken jointly, or by the husband, shows that the husband is seen as the main decision-maker about family planning. This has also been found in other studies in Sub-Saharan Africa [24–26]. In many countries in the region, use of family planning is, and traditionally has been, the husband’s decision. A woman who decides to use contraceptives on her own is seen as being disrespectful and promiscuous and could be suspected of having extra-marital affairs [18, 24]. This might explain why women are unwilling to take family planning decisions on their own, even though they have the rights and possibility to do so. However, arguments for the need to change this presumption of male dominance in decision-making were raised in this study as well as in previous research [18, 27], on the basis that women are the ones who suffer the risks of childbearing. Women in our study wanted to be independent in their own decision making about using contraceptive methods. This exercise of choice is described by Karp et al. to act on own preferences and requires confidence from the woman to initiate the discussion about family planning, negotiation with the partner and finally the woman’s decision-making [28]. To support women to reach own preferences it is important that healthcare professionals in family planning services take a key role in supporting women’s autonomous decision making about contraceptive use and should be careful not to undermine women’s confidence [27].

Another important finding in this study was that male involvement and education was desired from the women’s perspective. It might seem contradictory that in order to improve a woman’s power in her decision making, the husband needs to be involved. In societies with low gender equality and where men are socially dominant, men sometimes act as gatekeepers, restricting women’s access to sexual and reproductive information and services; moreover, gender inequality often leads to poor communication about sexual and reproductive health [21]. A male-involvement intervention study in Malawi [29] found that family planning uptake increased after the provision of tools to have difficult conversations and the implementation of male education about gender values in the household and decision-making as well as the methods and benefits of family planning. Women in that study felt more respected and involved in decisions about contraceptive use because of better communication. They [29] also suggested that an increased presence of the husband during labour, birth, and the postpartum period could ease the couple’s decision-making about postpartum family planning. This result is supported by a systematic review [30] which showed that male involvement during pregnancy and the postpartum period was associated with more benefits, such as an increased use of maternal health services (measured in terms of number of antenatal visits), compared to when they were involved only during labour. Since male opposition in our study along with others [20] was caused by gender norms based on lack of interest in contraception and a desire for many children, counselling and education addressing these problems are important.

Another interesting finding was that the women did not intend to live with their husbands during the early postpartum period. This could be explained both by religious practice and by the cultural tradition of practising sexual abstinence [31]. The need for contraceptives when returning to their husbands might be rooted in a lack of the possibility to decline sexual activity, as reported for around 25% of all reproductive-age women living in a relationship in Sub-Saharan Africa [32]. A previous study [11] reported that the average time gap between resuming sexual activity and starting to use contraception is 5 months in Tanzania. It can be argued that if healthcare and society aim to involve men, the discussion about family planning could start earlier between each couple and hence possibly limit the time spent without contraception and reduce the risk of an unintentional pregnancy.

### Methodological considerations

Credibility was established and strengthened by a well-considered method and the use of FGDs. It can be argued that individual in depths interviews could have generated more deep data at an individual level. However, FGDs create group dynamic and generate rich data [15]. The interviews were conducted by a native-speaking midwife to avoid language barriers and to achieve fluidity during the discussions. The interviewer’s familiarity with the culture, context, and society enabled her to understand the participants, and enabled the participants to feel more familiar with the situation. However, this might have caused some limitations in data depth. Since the culture was also rooted within the interviewer, follow-up questions on the cultural aspects of barriers were sometimes not asked. Despite this issue, the data collected were rich. It is worth noting that the findings about barriers to using postpartum family planning expressed by the women in this study are context-specific to a referral hospital in Zanzibar, and not necessarily true across regions in Zanzibar. Hence, different countries and settings interpret these findings with consideration of their own context.

## Conclusions and implications

This study reveals barriers to the use of postpartum family planning among women in Zanzibar: lack of knowledge about the methods, fear of side-effects, and limited power in decision-making about contraceptive use. To overcome these barriers, both in Zanzibar and in other settings with similar conditions, there is a need for high-quality counselling on contraceptive methods and how they work. This could mitigate fear and misconceptions about side-effects, and increase the understanding of how useful these methods are. To enhance women’s ability to use contraception postpartum and to increase participation in the decision-making, future family planning programs should work towards increased male involvement in family planning education and discussion. In a wider perspective it is essential to highlight the need for sexuality education and investment in woman’s education, in general, to enhance gender equality and woman’s agency to make decisions on their health. Based on the findings of this study, it is suggested that future research should focus on the involvement of men in family planning.

## Electronic supplementary material

Below is the link to the electronic supplementary material.

Appendix 1. Interview guide

## Data Availability

The datasets used and analysed during the current study are available from the corresponding author on reasonable request.

## List of abbreviations

FGD: Focus group discussion
