# Supplementary material for: Barriers to using postpartum family planning among women in Zanzibar, Tanzania
Source: BMC Womens Health. 2023 Apr 17;23:182. doi: 10.1186/s12905-023-02330-2 (PMC10111817; doi:10.1186/s12905-023-02330-2)
Supplement: Supplementary file 1 — Appendix 1. Interview guide [file 12905_2023_2330_MOESM1_ESM.docx]

## Appendix 1. Interview guide

**Main question:**
Why is postpartum family planning useful?

**Probing questions:**

1. What is not good about family planning?
2. Can you explain why?
3. From where do you mostly get your information?

**Main question:**
Given that you just gave birth, have you considered using family planning to delay the next pregnancy?

**Probing questions:**

1. If no, what are the reasons for not using family planning?
2. Please tell me more about that.
3. Is there anything or anyone that opposes usage of family planning?

**Main question:**What do you think is the general opinion about postpartum family planning among women in Zanzibar?

**Probing question:**

1. Can you explain what you mean by…?

**Main question:**
Do you know where to seek help if you want to use family planning? Is it easy to access?

**Probing questions:**

1. How does this affect you?
2. Do you feel like you have enough knowledge about postpartum family planning and your alternatives?

**Main question:**What could the family planning services do better in your opinion?

**Probing questions:**

1. Can you please give an example?
2. In what way would that help you?

**Main question:**

Is there anything else you would like to add?
